# Modified hood technique for single-port robot-assisted radical prostatectomy contributes to early recovery of continence

**DOI:** 10.3389/fsurg.2023.1132303

**Published:** 2023-05-03

**Authors:** Haoxun Zhang, Zikuan Ning, Guang Jia, Guoling Zhang, Jiuliang Wang, Hua Liu, Boju Tao, Chunyang Wang

**Affiliations:** ^1^ Department of Urology, First Affiliated Hospital of Harbin Medical University, Harbin, China; ^2^ Department of Operating Room, First Affiliated Hospital of Harbin Medical University, Harbin, China

**Keywords:** robot-assisted surgery, prostatectomy, incontinence, single-port surgery, extraperitoneal pathway

## Abstract

**Background and purpose:**

Urinary incontinence is one of the common side effects of robot-assisted radical prostatectomy (RARP). Here, we described the modified Hood technique for single-port RARP (sp-RARP) and assessed the interest of this new technique for early continence recovery.

**Methods:**

We retrospectively reviewed 24 patients who underwent sp-RARP modified hood technique from June 2021 to December 2021. The pre-and intraoperative variables, postoperative functional and oncological outcomes of patients were collected and analyzed. The continence rates were estimated at 0 day, 1 week, 4 weeks, 3 months and 12 months after catheter removal. Continence was defined as wearing no pad over a 24 h period.

**Results:**

Mean time of operation and estimated blood loss were 183 min and 170 ml, respectively. The postoperative continence rates at 0 day, 1 week, 4 weeks, 3 months and 12 months after catheter removal were 41.7%, 54.2%, 75.0%, 91.7% and 95.8%, respectively. There were two patients who detected positive surgical margins and no patients observed complications requiring further treatment.

**Conclusion:**

The modified hood technique is a safe and feasible method that provides better outcomes in terms of early return of continence, without increasing estimated blood loss and compromising oncologic outcomes.

## 1. Introduce

Prostate cancer is the world's second most frequent cancer, with the sixth highest fatality rate of all malignancies. Meanwhile, it is the most prevalent solid organ cancer in male. The most frequent clinical type of prostate cancer is localized prostate cancer, which accounts for more than 90% of cases at the time of diagnosis (1). In recent years, robotic-assisted radical prostatectomy (RARP) has increasingly become a surgical treatment option for localized prostate cancer worldwide, with reduced blood loss and faster postoperative recovery compared with open radical prostatectomy (ORP) and laparoscopic radical prostatectomy (LRP) (2, 3). Since Kouch first reported using single-port laparoscopy radical prostatectomy in 2008 (4), an increasing number of urologists are trying single-port robotic surgery and demonstrated that sp-RARP may have advantages in terms of shorter hospital stays and minimal postoperative pain compared to multi-port RARP (5–7).

However, almost all surgeries have a certain chance of adverse complications. The primary complications of radical prostatectomy are urinary incontinence and sexual dysfunction. Incontinence, in particular, can markedly impair the patients' quality of life (8). Therefore, several surgical techniques have been proposed to hasten continence recovery for patients after radical prostatectomy, including the preservation of the bladder neck and maximum length of the urethra, the nerve-sparing technique, preserving the puboprostatic ligament and endopelvic fascia, anterior reconstruction, Patel stitch, and total anatomical reconstruction for incontinence, etc (9–13).

The Hood procedure refers to a new surgical technique that retains the contents of the Retzius space utilizing an anterior approach, as proposed by Vinayak G et al., preserving the anatomical structures associated with urinary control and erectile function, enabling early recovery of continence (14). However, due to the limited space for surgical operation of this surgical procedure and the easy damage to the bladder and ureter during the operation, Retzius-sparing RARP is not recommended, especially for prostate cancer with late clinical-stage, so its clinical application is limited (15).

The modified-Hood technique presented in this study retains the intrafascial neurovascular bundle (NVB) and part of the Retzius space by a single-port laparoscopy extraperitoneal route, which preserved the periurethral structure including dorsal vein complex (DVC) at the maximum extent for early return of urinary continence. The preserved periurethral tissues include the two sides pelvic fascia of the distal prostate, arcus tendinous, detrusor apron, puboprostatic ligament, DVC, and partial urethral supporting structures, exhibiting a “hood” appearance, similar to the structure preserved by Vinayak G et al.

This study aims to present a novel modified Hood technique for single-port robot-assisted radical prostatectomy and evaluate the recovery of urinary continence undergoing this technique.

## 2. Materials and methods

### 2.1. Study participants

This retrospective study involved 24 patients diagnosed with prostate cancer under ultrasound-guided prostate biopsy and operated on by an experienced surgeon (C.Y.W) at a single medical center from June 2021 to December 2021. Surgical planning was discussed with each patient and informed consent was obtained before surgery. Inclusion criteria were patients with localized or locally advanced prostate cancer without distant metastases, and with a life expectancy of more than 10 years. Exclusion criteria were patients with biopsy Gleason score ≥8, PSA ≥ 20 and mp-MRI showed that the tumor invaded one side of prostate extracapsular tissues (most important) at the same time. Moreover, patients with neurogenic bladder, prior prostate treatment, previous urinary stricture and incontinence history, multiple metastases, and incomplete clinical data were also excluded from this study. The study was approved by the ethics committee of the First Affiliated Hospital of Harbin Medical University (NO.IRB-AF/SC-04/02.0).

### 2.2. Surgical technique

All patients were operated on by an experienced surgeon using an extraperitoneal approach through the DaVinci Xi platform. We described the main surgical steps of the modified Hood technique for sp-RARP.

#### 2.2.1. Trocar placement

**Figure 1:**
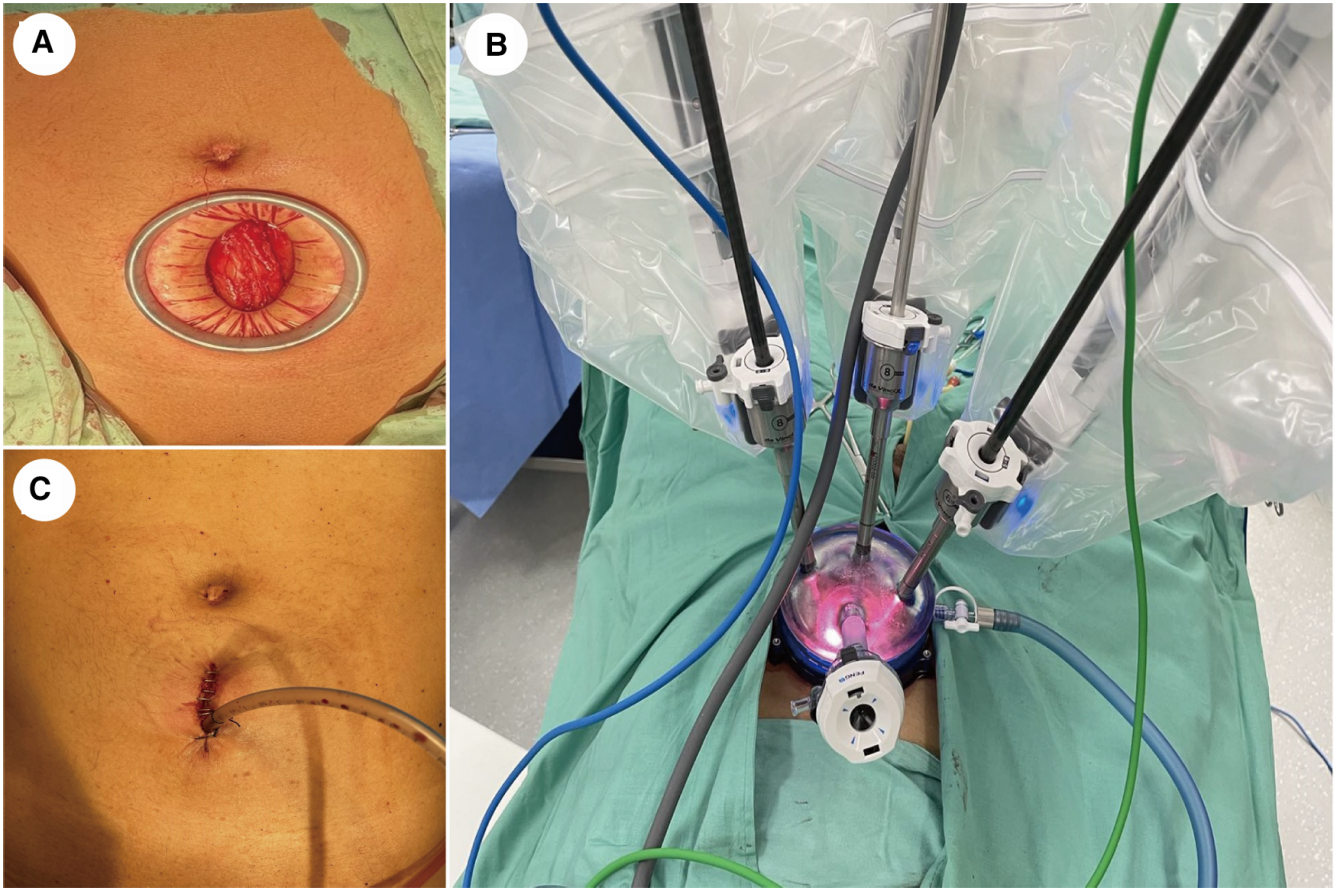
Abdominal incision and port placement. (**A**) A 3−5 cm vertical incision was made with a wound protector approximately 5 cm above the pubic symphysis. (**B**) A quadrichannel laparoscopic port was installed with two robotic arms, a high-definition laparoscope facing 30-degree up, and an assistant's port. (**C**) The drainage tube was placed in the same incision.

An extraperitoneal approach was used for all patients who were placed in a 10°to 20°Trendelenburg position after general anesthesia. A 3–5 cm vertical skin incision was made approximately 5 cm above the pubic symphysis. After incision of the anterior rectus fascia and separation of the rectus abdominis, a dilator made of an inflated surgical glove and a disposable urinary catheter was used to develop additional extraperitoneal space. A 100 mm multi-channel laparoscopic port was placed through the incision (Figure 1).

#### 2.2.2. Extraperitoneal space creation

Maryland bipolar forceps and 8-mm monopolar scissors were utilized to remove the fat on the surface of the prostate and the anterior bladder neck. During this period, pay attention to protecting the tendon arch and the puboprostatic ligament from being cut off.

#### 2.2.3. Bladder neck transection

**Figure 2:**
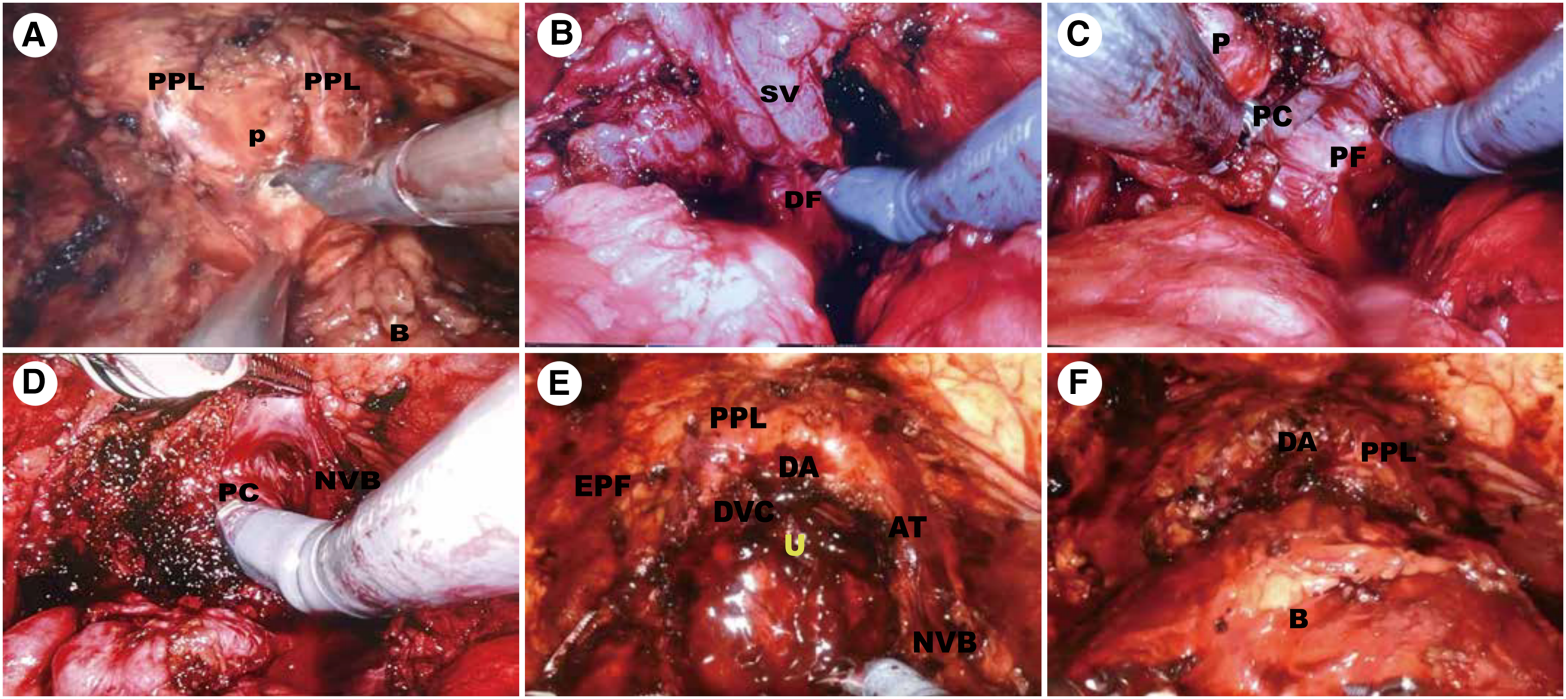
Surgical technique. (**A**) Preservation of the Endopelvic Fascia and Bladder neck transection. (**B**) Dissection of the Vas Deferens and Seminal Vesicles. (**C,D**) Complete Intrafascial NVB sparing Technique. (**E**) Development of hood structure. (**F**) Vesicourethral anastomosis. P, prostate; B, bladder; U, urethra; PPL, puboprostatic ligaments; AT, arcus tendineus; EPF, endopelvic fascia; SV, DF, Denonvillier fascia; PC, prostatic capsule; PF, prostatic fascia; NVB, neurovascular bundle; DVC, deep venous complex; DA, detrusor apron.

The position of the prostatovesical junction was determined by observing the outline of the prostate from the side, indenting or pinching the bladder, and pulling the catheter. A transverse incision was made at the midline of this position to expose the catheter, and the posterior wall of the urethra was dissected after the catheter was withdrawn. At the bladder neck's 5–7 o'clock position, the posterior bladder neck is further incised down to the longitudinal fibrous layer and the posterior deltoid layer of the bladder neck (Figure 2A).

#### 2.2.4. Dissection of seminal vesicle and vas deferens

The fibrous layer was cut transversely to expose the vas deferens and seminal vesicles underneath. Seminal vesicles were dissected to the tip along the avascular plane formed by the fascia surrounding the seminal vesicles (Figure 2B).

#### 2.2.5. NVB sparing techniques

The seminal vesicles and vas deferens were lifted and the Denovilliers' fascia was pulled cephalad (Figure 2C). By a combination of sharp and blunt dissection, a plane was developed between the prostatic capsule and fascia. We separated the prostate dorsally towards the apex and this dissection was continued intrafascially to preserve the NVB (Figure 2D).

#### 2.2.6. Development of hood structure and urethral dissection

The ventral plane of the prostate was also dissected close to the prostate capsule, retaining the detrusor apron structure located on the ventral side of the prostate. Then, we dissected the apex of the prostate using monopolar scissors and Maryland bipolar forceps were used to coagulate the bleeding vessels. When the urethra was fully dissociated, the urethra was cut off close to the apex of the gland (Figure 2E).

#### 2.2.7. Vesicourethral anastomosis

Mesh vesicourethral anastomosis is performed using two separate barbed sutures. The posterior wall of the bladder neck was lifted, and a barbed suture was used at the 5 o'clock position to pass from outside to inside through the posterior wall of the bladder neck and from inside to outside through the posterior wall of the urethra and the posterior median ridge. Another barbed suture is anastomosed to the body of the bladder along the vesicourethral anastomosis (Figure 2F).

### 2.3. Data collection and analysis

Baseline patient characteristics, intraoperative variables, and postoperative functional and oncological outcomes were collected and analyzed. Complications were accessed using the Clavien-Dindo classification. Pain scores on postoperative day 1 were used as pain outcomes. The postoperative pain was assessed by VAS (visual analogue scale) score. The pain scores ranged from zero to ten and were recorded by the house surgeon. The postoperative day 1 pain score was the first pain score recorded on postoperative day 1 and the patients with score from 0 to 3 were defined as pain-free. The postoperative urinary continence was assessed by counting the number of pads for 24 h in patients with urinary incontinence at 0 day, 1 week, 4 weeks, 3 months, and 12 months after catheter removal. Patients were defined as continence if they wore no pad over a 24 h period. Biochemical recurrence was defined as two consecutive increases in PSA readings of >0.2 ng/ml, according to American Urological Association recommendations.

Baseline patient characteristics were collected during the hospitalization, including age, body mass index (BMI), prior abdominal surgery, prostate volume, prostate-specific antigen (PSA) level, and biopsy Gleason score. The preoperative functional characteristics were assessed using the International Prostate Symptom Score (IPSS). Perioperative and postoperative follow-up variables involved operative time (from skin incision to skin closure), estimated blood loss (EBL), transfusion rate, lymphadenectomy, intraoperative complications, postoperative pain, postoperative Gleason score, positive surgical margin (PSM) rates, indwelling catheter days, and hospitalization days.

Categorical data were given as percentages, whereas continuous variables were presented as the median and interquartile range (IQR). SPSS 22.0 was used for all statistical analyses.

## 3. Results

Table 1 summarizes the baseline demographic and clinical data of the 24 patients involved in this study. The median age, BMI, prostate volume, total and Percent free PSA level of 24 patients at the time of surgery were 70 years (IQR 64.50–76.50), 24.85 kg/m2 (IQR22.70–26.80), 34.88 ml (IQR24.06–62.05), 16.98 ng/ml (IQR3.49–36.41) and 0.14 (IQR0.08–0.48), respectively. The median IPSS score was 15(IQR13–22) and was used to assess the preoperative functional parameters of patients. In addition, there were five patients possessing the history of abdominal surgery.

**Table 1:** Patients’ characteristics and preoperative data.

Variables	Overall population
Age, median (IQR), year	70.00 (64.50–76.50)
BMI, median (IQR), kg/m^2^	24.85 (22.70–26.80)
Prior abdominal surgery, *n* (%)	5 (20.83)
Prostate volume median (IQR), ml	34.88 (24.06–62.05)
Total PSA, median (IQR), ng/ml	16.98 (3.49–36.41)
Percent free PSA, median (IQR)	0.14 (0.08–0.48)
cT stage, *n* (%)	
cT1	6 (25.00)
cT2	9 (37.50)
cT3a	4 (16.67)
cT3b	5 (20.83)
Biopsy gleason score, *n* (%)	
6	3 (12.50)
3 + 4	6 (25.00)
4 + 3	7 (29.17)
8–10	8 (33.33)
IPSS score, median (IQR)	15 (13–22)
D’ Amico risk group, *n* (%)	
1	3 (12.50)
2	12 (50.00)
3	9(37.50)

IQR, interquartile range; BMI, body mass index; PSA, prostate specific antigen; IPSS, International Prostate Symptoms Score.

The perioperative and pathological results were presented in Table 2. The median operative time and blood loss were 182.5 min (IQR141.0–208.3) and 170 ml (IQR25–300), respectively. A total of twenty-one patients with the Gleason score ≥7 were performed lymph node dissection. And nine patients were pain-free on the first day after the surgery with the VAS score from 0 to 3. The final pathology report revealed three patients with Gleason score of 3 + 3, seven patients with Gleason score of 3 + 4, five patients with Gleason score of 4 + 3 and nine patients with Gleason score of 8–10. A total of two patients' surgical margins were positive. With regard to complications, there were no patients who observed complications requiring further treatment, such as lymphatic cysts and hematomas.

**Table 2:** Comparison of perioperative and histopathologic data.

Variables	Overall population
Perioperative	
Operative time, median (IQR), min	182.5 (141.0–208.3)
Estimated blood loss, median (IQR), ml	170.0 (25.0–300.0)
Transfusion rate, *n* (%)	0
Lymphadenectomy, *n* (%)	21 (87.50)
Indwelling catheter time, median (IQR), d	5 (3–10)
Hospitalization duration, median (IQR), d	6 (3–11)
Complications, *n* (%)	
Clavien I–II	3 (12.50)
Clavien III–V	0
Postoperative pain assessment, *n* (%)	
VAS score 0–3	9 (37.50)
VAS score 4–6	13 (54.17)
VAS score 7–10	2 (8.33)
Pathologic	
Pathologic stage (*n*)	
pT2	11 (38.46)
pT3a	6 (38.46)
pT3b	7 (23.08)
Pathologic gleason score (*n*)	
6	3 (12.50)
3 + 4	7 (29.17)
4 + 3	5 (20.83)
8–10	9 (37.50)
Positive surgical margins (*n*)	2 (8.33)
6-mo biochemical recurrence, *n* (%)	2(8.33)

Table 3 showed that the outcome of postoperative urinary continence recovery. According to the previously described definition, ten patients gain urinary continence after catheter removal at 0 day, thirteen patients at 1 week, eighteen patients at 4 weeks, twenty-two patients at 3 months and twenty-three patients at 12 months.

**Table 3:** Continence data at various follow-up points.

Time	Overall population
0 day	10 (41.67)
1 week	13 (54.17)
4 weeks	18 (75.00)
3 months	22 (91.67)
12 months	23 (95.83)

**Table 4:** Comparison of pre-, intra-, and postoperative variables between the modified hood technique, DVC-ligation technique published by Ilter Tüfek et al and hood technique published by Vinayak G et al.

Study authors	Vinayak G et al	Ilter Tüfek et al	Our series
*n*	300	50	24
Age, median (IQR), year	64 (58–68)	61.75	70.00 (64.50–76.50)
BMI, median (IQR), kg/m^2^	27 (25–29)	26.48	24.85 (22.70–26.80)
Prostate volume median (IQR), ml	51 (40–64)	41.94	34.88 (24.06–62.05)
Total PSA, median (IQR), ng/ml	6 (4–8)	7.1	16.98 (3.49–36.41)
Operative time, median (IQR), min	169 (147–195)	167	182.5 (141.0–208.3)
Estimated blood loss, median (IQR), ml	150	185	170.0 (25.0–300.0)
Positive surgical margins (*n*)	10	2	2
Definition of continence	No Pads/24 h	No Pads/24 h	No Pads/24 h
Urinary continence	1 week: 21%	1 week: NA	1 week: 54.2%
	1 month: 83%	1 month: 74%	1 month:75.0%
	3 months: 91%	3 months: 90%	3 months: 91.7%

## 4. Discussion

Because of better postoperative oncological and functional results, robot-assisted radical prostatectomy (RARP) has gradually replaced traditional radical prostatectomy as the main surgical treatment for clinically localized or locally advanced prostate cancer (16). Urinary incontinence is one common dreaded complication that significantly affects patients' quality of life who underwent RARP, which merits further investigation. It is generally accepted that maximum preservation of pelvic original anatomy structure is essential to the recovery of incontinence (17). Over the past several years, we had attempted several techniques, including reconstruction of the posterior aspect of the striated sphincter, bladder neck preservation, neurovascular bundles preservation, DVC ligation-free, periurethral suspension stitch, lateral prostatic fascia preservation, full functional-length urethral sphincter preservation to reduce postoperative incontinence. Based on our extensive experience with robotic surgery and exploration of improved urinary continence techniques, we proposed a modified hood technique, which combines the advantages of single-port laparoscopy, extraperitoneal robotic surgery, and hood technique to make patients suffer less trauma and return continence sooner.

Conventional RARP often requires multiple surgical orifices for operation, which causes pain in multiple areas for patients after surgery and poor cosmetic results of the abdominal surgical scar. Single-port RARP requires only one incision of about 3–5 cm with good incision healing and cosmetic results. A Comparison between single-port and multiport robot-assisted prostatectomy conducted by Vigneswaran showed that there were significant differences in pain-free on the first postoperative day (sp-RARP vs. mp-RARP, 30% vs. 12%, *p* = 0.004) and hospital stays (sp-RARP vs. mp-RARP, 1 vs. 2, *p* = 0.002) and demonstrated the safety and feasibility of SP-RARP (5).

Vinayak G et al. first introduced the Hood technique, a modified anterior approach to preserve the contents of the space of Retzius, in 2020. The Retzius-sparing technique preserved the structure around the urethra, so that there was fixed support around the anastomosis, to avoid the contraction tension of the detrusor muscle directly acting on the anastomosis, which contributed to achieve better urinary continence after surgery. The study showed that urinary continence rates of hood technique at 1 week, 1 month, and 3 months after catheter removal were 21%, 83%, and 91%, respectively and the positive surgical margin rate was 6% (14).

Our technique retains NVB and part of the Retzius space by an extraperitoneal route and compared with the traditional hood, the advantage is that the vision is clearer, the steps are simpler, and the suspension support structures around the retropubic urethra can be preserved to the greatest extent. In addition, the extraperitoneal route does not interfere with intra-abdominal organs, resulting in a lower incidence of anesthesia and bowel-related complications, and faster postoperative recovery.

In our study, the urinary continence rates in the modified hood technique group were 54.2%, 75.0%, and 91.7% at 1 week, 1 month, and 3 months after catheter removal, which is significantly higher than the Vinayak G and colleagues' percentages of 21% at 1 week (Table 4). Positive surgical margins were important risk factors for residual lesions and recurrence, resulting in potential side effects for patients (18). Positive margin rates for PT2 carcinomas ranged from 11% to 25%, and 36% to 47% for PT3 carcinomas in other investigations using the Retzius-Sparing technique (19–22). In our present study, the overall positive surgical margin rates were 8.33%, slightly higher than the 6% of Vinayak G.

Moreover, it should be pointed out that we have adopted DVC ligation-free in our technology. Suture ligation of the DVC is generally accepted worldwide as an effective method to reduce estimated blood, but it may injure the external sphincter and decrease the functional urethral length (23). Recently, many scholars explored optimal control of the DVC in RARP, including suture ligation followed by athermal DVC division (SL-DVC), athermal DVC division followed by selective suture ligation (DVC-SSL), and DVC ligation-free techniques (24–26). Ilter Tüfek et al. confirmed that the DVC ligation-free technique was a feasible technique in RARP that significantly shortened operative time (146.8 vs. 178.4 min, *P* = 0.0005), did not increase the estimated blood loss (185 vs. 184.2 ml, *P* = 0.92) and contributed to quicker recovery of continence (90% vs. 74%, *p* = 0.06) (27). In our study, before dissecting the apex of the prostate and cutting off the urethra, we momentarily elevated the pneumoperitoneal pressure, cut off the DVC with monopolar scissors, and then coagulated the DVC with Maryland bipolar forceps. The DVC, which is a venous plexus and comprises smooth muscle tissue, has been reported to have a mean width, height, and area of 2.0 ± 0.4 cm, 1.2 ± 0.3 cm, and 1.8 ± 0.6 cm^2^, respectively (28). The diameter of vessels that could be coagulated by bipolar forceps reaches approximately 5 mm and the diameter of a single vessel may be within the coagulable range. In our study, all 24 surgeries were completed and there was no significantly difference in estimated blood loss between our series and Ilter Tüfek and Vinayak G (170 vs. 185 vs. 150 ml).

It is necessary to acknowledge some of the study's limitations. First, the fact that small sample size and a single-center retrospective study should be acknowledged; second, assessment of urinary continence by the number of pads may be influenced by the subjective judgment of patients; third, longer follow-up period for functional and oncological outcomes are needed to confirm our results.

## 5. Conclusion

The novel modified hood technique for sp-RARP appears safe and effective, contributing to early continence recovery without increasing estimated blood loss and compromising oncologic outcomes. However, our findings need to be validated further in prospective randomized trials or a large sample.

## Data Availability

The raw data supporting the conclusions of this article will be made available by the authors, without undue reservation.
